# Poster Session II - A301 PREVALENCE AND EPIDEMIOLOGICAL FEATURES OF ADULT UPPER GASTROINTESTINAL CROHN’S DISEASE OVER THE LAST 30 YEARS: A SCOPING REVIEW

**DOI:** 10.1093/jcag/gwaf042.301

**Published:** 2026-02-13

**Authors:** D Lupas, N Natt, P Taylor, Z Mansoor, Y Yuan, R Sedano

**Affiliations:** Medicine, Western University Schulich School of Medicine and Dentistry - Windsor Campus, Windsor, ON, Canada; London Health Sciences Centre, London, ON, Canada; University Health Network, Toronto, ON, Canada; London Health Sciences Centre, London, ON, Canada; London Health Sciences Centre, London, ON, Canada; London Health Sciences Centre, London, ON, Canada

## Abstract

**Background:**

Crohn’s disease (CD) is an inflammatory bowel disease (IBD) that can affect any part of the gastrointestinal tract. Although upper gastrointestinal (UGI) manifestations are well recognized in pediatrics, their frequency and clinical relevance in adults have historically been underestimated. Challenges include selective use of endoscopy, misattribution of UGI lesions to other conditions, and inconsistent application of classification systems. These limitations have led to highly variable prevalence estimates and hindered understanding of UGI disease in adult CD.

**Aims:**

To summarize the prevalence of UGI-CD in adult patients with CD, understand demographic variations in the presentation of UGI-CD, and summarize current knowledge gaps.

**Methods:**

We conducted a scoping review of MEDLINE, Embase, and Cochrane CENTRAL (1995–Nov 2024) following JBI methodology and PRISMA-ScR guidance. Eligible studies reported adult UGI-CD prevalence or related data. Extracted variables included study characteristics, prevalence estimates, site-level involvement, demographics, and disease behaviour. Systematic reviews were also included, providing pooled prevalence estimates and insights into ethnic variation, phenotype, and outcomes.

**Results:**

A total of 115 primary studies and seven systematic reviews were included. Across 95 adult cohorts with extractable prevalence data, the median prevalence of UGI-CD was 9.1% (IQR 4.5–14.7%). Higher rates were consistently observed in cohorts using systematic endoscopy and biopsy at diagnosis regardless of symptomatology, while population-based and retrospective cohorts reported lower values. Isolated UGI-CD was rare, with a median prevalence of 1.37% (IQR 0.84–3.42%). Prevalence varied geographically and ethnically, with lowest rates in South Asian cohorts and higher rates in East Asian and Middle Eastern cohorts. Site-specific reporting (esophageal, gastric, duodenal, jejunal) was inconsistent, and few studies detailed modifiers such as smoking, family history, or extraintestinal manifestations.

**Conclusions:**

Adult UGI-CD is more common than previously recognized, but estimates remain highly variable due to inconsistent definitions and heterogeneous detection methods. To move the field forward, studies must adopt standardized, site-specific adult definitions and systematic diagnostic protocols, including clear guidance on when to perform endoscopy with biopsy, to generate accurate prevalence estimates and enable valid cross-study comparisons.

**Funding Agencies:**

None

